# Parental care masks a density-dependent shift from cooperation to competition among burying beetle larvae

**DOI:** 10.1111/evo.12615

**Published:** 2015-03-21

**Authors:** Matthew Schrader, Benjamin J M Jarrett, Rebecca M Kilner

**Affiliations:** 1 Department of Zoology, University of CambridgeCambridge, CB2 3EJ, United Kingdom

**Keywords:** Burying beetle, *Nicrophorus vespilloides*, offspring size and number, parental care, sibling rivalry, trade-offs

## Abstract

Studies of siblings have focused mainly on their competitive interactions and to a lesser extent on their cooperation. However, competition and cooperation are at opposite ends on a continuum of possible interactions and the nature of these interactions may be flexible with ecological factors tipping the balance toward competition in some environments and cooperation in others. Here we show that the presence of parental care and the density of larvae on the breeding carcass change the outcome of sibling interactions in burying beetle broods. With full parental care there was a strong negative relationship between larval density and larval mass, consistent with sibling competition for resources. In the absence of care, initial increases in larval density had beneficial effects on larval mass but further increases in larval density reduced larval mass. This likely reflects a density-dependent shift between cooperation and competition. In a second experiment, we manipulated larval density and removed parental care. We found that the ability of larvae to penetrate the breeding carcass increased with larval density and that feeding within the carcass resulted in heavier larvae than feeding outside the carcass. However, larval density did not influence carcass decay.

Biological families have become microcosms for studying the evolutionary tension between cooperation and conflict (Parker et al. 2002). In sexually reproducing organisms, male and female parents must cooperate to create offspring and in many species parents work together to successfully rear these young. On the other hand, there is often intense conflict between males and females over aspects of mating and how much each parent should invest in rearing young (Arnqvist and Rowe 2005; Lessells 2012). Similarly, interactions between parents and offspring can be viewed through the lenses of cooperation and conflict. Parents of many species provision their young and offspring may honestly communicate their need for parentally supplied resources (Rauter and Moore 1999; Kilner and Hinde 2008; Mas and Kolliker 2008). However, parents and offspring are usually not genetically identical to one another and this genetic asymmetry generates an evolutionary conflict of interest, with offspring selected to demand more resources from their parents than is optimal for parents to supply (Trivers 1974).

Interactions between dependent siblings may also represent a tension between cooperation and conflict (Roulin and Dreiss 2012). Conflict between dependent siblings (i.e., sibling rivalry) is an obvious and often brutal part of family life for many animals. Indeed, offspring of many species display morphological or behavioral traits that are probable adaptations for competing with their siblings (Mock and Parker 1997). Cooperation between dependent siblings is much less obvious and has received scant attention compared to sibling competition. Nevertheless, there are some examples suggesting that dependent siblings engage in cooperative behaviors. For example, barn owl (*Tyto* alba) nestlings have been documented feeding one another (Marti 1989) and there is evidence that barn swallow (*Hirundo rustica*) nestlings moderate their selfishness when their siblings have been food deprived (Romano et al. 2012). Although not completely dependent on parental care, nymphs of the European earwig, *Forficula auricularia* also appear to share food through allo-coprophagy (feeding upon frass released by brood mates) or proctodeal trophallaxis (mouth to anus feeding) and this sharing increases the amount of weight gained by interacting siblings (Falk et al. 2014). Siblings may also engage in behaviors that may not have evolved as adaptations for cooperation per se but that still benefit one another. These behaviors have been referred to as mutually beneficial (West et al. 2007). One example of such a mutually beneficial behavior is the effect of siblings on the thermal environment experienced by nestling birds and mammals. Here the mere presence of nest or littermates enhances the thermal environment experienced by each sibling, which increases survival and offspring performance (Forbes 2007; Hudson and Trillmich 2007).

Competition and cooperation are at opposite ends on a continuum of potential sibling interactions (Forbes 2007). The precise position on this continuum of any given sibling interaction is likely to vary, with ecological factors tipping the balance toward competition in some environments and cooperation in others. Such a shift commonly occurs in interactions between plant species, from competition in benign environments to facilitation in stressful environments (He et al. 2013). There is also evidence that stressful environments can induce cooperation between different individuals of the same species. For example, in European rabbits (*Oryctolagus cuniculus*) soil temperature appears to determine the relative importance of competition and mutual benefit among sibling pups (Rödel et al. 2008). When temperatures are warm there is a negative relationship between litter size and pup growth rate and this trade-off likely reflects sibling competition for milk. However, when temperatures are below 10°C, this relationship becomes nonlinear with the highest pup growth rates occurring in litters of three. Rödel et al. (2008) suggest that the thermal benefits to pups of being in large broods outweigh the negative impact of sibling rivalry for milk but only when temperatures are low and not when they are high.

Here we use data from an experiment involving burying beetles, *Nicrophorus vespilloides*, to uncover a density-dependent shift between mutually beneficial and competitive interactions among sibling larvae that are masked by the presence of parental care. We manipulated the presence of posthatching parental care (full parental care or no posthatching care) and measured larval density (the number of larvae at dispersal divided by the mass of the breeding carcass) and mean larval mass at dispersal for each brood. With full parental care, there was a strong negative relationship between larval density and average larval mass, consistent with the presence of sibling competition for resources when parents are present. In the absence of parental care, initial increases in larval density had beneficial effects on average larval mass but further increases in larval density reduced larval mass. This nonlinear relationship between larval density and larval mass likely reflects a density-dependent shift from mutually beneficial to competitive larval interactions. We complemented this experiment with another experiment designed to elucidate the nature of mutually beneficial interactions between burying beetle larvae. This experiment investigated whether offspring assist each other in penetrating the carcass, a first key step in acquiring resources. We also considered whether the antimicrobial secretions produced by larvae (Arce et al. 2013; Reavey et al. 2014) mean that greater numbers are more effective at defending the carcass from microbial competitors.

## Methods

### STUDY SPECIES

Like all species in the genus, *N. vespilloides* breeds on the carcasses of small vertebrates. Upon encountering a carcass, parents mate and prepare the carcass for their young to feed upon. Carcass preparation involves shaving the fur or feathers from the carcass, rolling it into a ball, and smearing the surface of the flesh with oral and anal exudates that delay decomposition (Scott 1998). The eggs, which are laid near the carcass, hatch into altricial larvae that migrate to the carcass where they feed. *Nicrophorus vespilloides* larvae are capable of self-feeding, but are also provisioned by their parents with regurgitated predigested carrion. Although parental provisioning is facultative in *N. vespilloides*, offspring beg for parentally supplied resources and measures of breeding success and larval performance are higher when parents are allowed to provision larvae than when they are not (Eggert et al. 1998).

### EXPERIMENT 1: PARENTAL CARE AND THE RELATIONSHIP BETWEEN LARVAL DENSITY AND AVERAGE LARVAL MASS

The beetles used in this experiment were descended from field-collected beetles trapped in 2012 and outbred for five generations to create a stock population. In June 2013, we bred 400 pairs of beetles, 160 pairs were assigned to the full parental care treatment (Full Care) and 240 pairs were assigned to the no posthatching parental care treatment (No Care). We placed each pair in a box with soil and a thawed mouse carcass (8–14 g). These boxes were then put in a dark cupboard to simulate underground conditions. In the Full Care treatment, we allowed both parents to remain in the breeding box until larval dispersal (eight days after pairing). On the eighth day, we counted and removed all of the larvae from each breeding box, weighed each brood, and then calculated the average mass of larvae in each brood (total brood mass/brood size). We also calculated the density of larvae at dispersal (larval density) as the brood size at dispersal divided by the mass of the breeding carcass. Pairs assigned to the No Care treatment were treated identically, except we prevented parents from provisioning larvae by removing both parents from the breeding box after carcass preparation and egg laying but before the eggs had hatched (53 h after pairing, following Boncoraglio and Kilner [2012]).

Compared to the No Care treatment, there were very few Full Care broods with low larval densities. The lack of low-density broods in the Full Care treatment is not surprising because parental care increases larval survival and thus brood size (Eggert et al. 1998; Schrader et al. 2015). However, we were concerned that a relative lack of low-density broods in the Full Care treatment might influence our estimate of the relationship between larval density and mean larval mass. Therefore, to increase our sample of low-density Full Care broods, we experimentally reduced brood size in a sample of 38 families. To create these experimentally reduced broods, we first bred pairs of beetles following the protocol used in the Full Care populations. We next randomly assigned each pair of beetles a manipulated brood size of between two and nine larvae. We then removed the parents’ entire brood from the breeding carcass one day after hatching and returned the assigned number of larvae to the carcass. The manipulated brood and both their parents were then placed in a new breeding box and returned to the cupboard where they remained until larval dispersal. We then collected data on larval density and average larval mass as described above. Note that the goal of this manipulation was to increase the number of small broods in the Full Care treatment. We decided not to conduct a similar manipulation in the No Care treatment for two reasons. First, there were already several No Care broods with low larval densities. Second, our experiments and those of other groups (Eggert et al. 1998; Schrader et al. 2015) have found that removing posthatching parental care reduces brood size in *N. vespilloides*. Thus experimentally reducing brood size in the absence of posthatching care was likely to result in high rates of complete brood loss.

To analyze the relationship between larval density and average larval mass in the Full Care and No Care treatments, we used linear and polynomial (quadratic and cubic) regression models. These models excluded pairs that failed to produce at least one dispersing larva. We compared the fit of each model to the simpler model (e.g., quadratic models were compared to linear models and cubic models were compared to quadratic models) and removed terms with *P* values < 0.05 from each model sequentially to obtain a minimal model for each treatment. We performed this analysis twice excluding and including the experimentally reduced broods.

To complement the descriptive analysis described above, we examined the effect of parental care, larval density, larval density^2^, and larval density^3^ on average larval mass using a general linear model (GLM) with a Gaussian error. We included in this analysis interactions between parental care and each larval density term (care × larval density, care × larval density^2^, and care × larval density^3^). These interactions terms test whether the slope (care × density) or curvature (care × density^2^, care × density^3^) of the relationship between larval density and mean larval mass differs between the Full Care and No Care treatments. We performed this analysis twice, excluding and including the experimentally reduced broods and removed interaction terms that were not statistically significant (*P* < 0.05).

### EXPERIMENT 2: THE EFFECT OF LARVAL DENSITY ON THE ABILITY OF LARVAE TO ENTER THE CARCASS AND CARCASS DECOMPOSITION

We conducted a brood manipulation experiment to test whether, in the absence of posthatching parental care, the ability of larvae to penetrate the breeding carcass and carcass decay were each affected by larval density. To do this, we first bred 80 pairs of beetles from our stock population following the same methods described above for the No Care treatment (i.e., we removed parents from each breeding box 53 h after pairing). Seventy hours after pairing the adults, we inspected each box for the presence of recently hatched first-instar larvae. Boxes containing larvae (*n* = 54) were paired at random and within each pair, one box was randomly assigned to the low larval density treatment and the other to the high larval density treatment. For each pair of boxes, we removed the prepared carcasses, gently removed all of the larvae from each carcass, and placed all of the larvae on the surface of a moist paper towel (larvae from both broods were pooled). After removing the larvae, we inspected each carcass for the presence of holes and noted when these were discovered. In a few cases, we discovered first-instar larvae within these holes that we had previously overlooked. These larvae were also gently removed and added to the pooled group of larvae on the paper towel. We then placed each carcass (now cleared of larvae) in a new breeding box filled one-third with moist soil and then added larvae back to the carcass. Boxes assigned to the low-density treatment were given 10 larvae and boxes assigned to the high-density treatment were given 20 larvae. When adding larvae, we placed them directly on top of the carcass.

After adding the appropriate number of larvae to each carcass, we returned the breeding boxes to the dark breeding cupboard. The next day (90 h after pairing) we inspected each breeding box and recorded whether larvae had penetrated the carcass, and whether there was any evidence of mold on the surface of the carcass. These inspections were made at four time points: 90, 93, 96, and 114 h after pairing. At 120 h after pairing, we inspected each breeding box for the presence of larvae feeding inside or outside of the carcass. We then removed the living larvae from these boxes, weighed the entire brood on a microbalance, and calculated the average larval mass for each brood.

We compared the proportion of cases in which larvae penetrated, and were feeding within, the carcass between the low- and high-density treatments using a chi-squared test. This analysis excluded replicates (*n* = 10) that had a hole in the carcass prior to the brood manipulation (i.e., at 70 h after pairing). All of the cases of larvae feeding within the carcass were discovered at 90 h and no more were discovered in subsequent censuses. Thus, our analysis of carcass penetration was restricted to the 90-h time point. We also compared the proportion of cases in which the breeding carcass began to mold between the low- and high-density treatments using a chi-squared-test. The proportion of carcasses with evidence of mold increased across time points so we analyzed the data from the first and last time points separately (i.e., the 90- and 114-h time points).

## Results

### EXPERIMENT 1: PARENTAL CARE AND THE RELATIONSHIP BETWEEN LARVAL DENSITY AND AVERAGE LARVAL MASS

**Figure 1:**
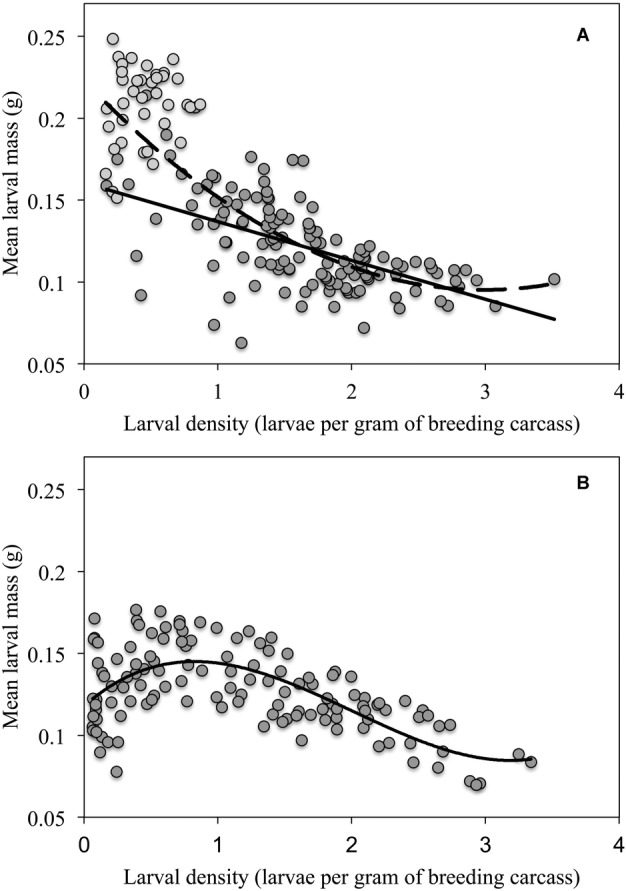
The relationship between larval density (number of larvae per gram of carcass) and mean larval mass in the Full Care (A) and No Care (B) treatments. For the Full Care treatment, we present data for both unmanipulated broods (dark gray circles) and experimentally reduced broods (light gray circles). Lines are linear or polynomial regression lines. For the Full Care treatment, we present regression lines excluding and including the experimentally reduced broods (solid and dashed lines, respectively).

The presence of posthatching parental care influenced the shape of the trade-off between larval density and average larval mass, because it decreased monotonically in the Full Care treatment but not in the No Care treatment (Fig.1). To compare the shape of this trade-off more precisely, we began by determining whether it was best described by a linear, quadratic, or cubic relationship in each case. In the Full Care treatment, there was a negative relationship between average larval mass and larval density that was best described by a linear regression model (Fig.1A, Table 1). When we supplemented these data with data from experimentally reduced broods, the relationship between average larval mass and larval density was best described by a quadratic regression model with a positive quadratic term. Thus the slope of the relationship between average larval mass and larval density declined as density increased (Fig.1A, Table 1). In both cases, larval mass was highest at the lowest larval density. By contrast, in the No Care treatment the relationship between average larval mass and larval density was best described by a cubic regression with a negative quadratic term (Fig.1B, Table 1). In the No Care treatment, larval mass was maximized at larval densities of approximately 0.77 larvae per gram of carcass.

**Table 1:** Best-fit regression models of mean offspring mass on larval density (*X*) for the Full Care (FC) and No Care (NC) populations in the first generation of the experiment

Care	*X*	*X*^2^	*X*^3^	*P*	*R*^2^
FC (*n* = 131)	−0.024 (± 0.0060)			<0.0001	0.26
FC with reduced broods (*n* = 169)	−0.086 (± 0.011)	0.014 (± 0.0036)		<0.0001	0.57
NC (*n* = 125)	0.072 (± 0.016)	−0.055 (± 0.012)	0.0092 (± 0.0026)	<0.0001	0.43

For each population, we present the parameter values (±SE), significance level, and *R*^2^ for the best-fit regression model. For the Full Care population, we present the best-fit regression models excluding (above) and including (below) experimentally reduced broods.

Next, we analyzed the differences between care treatments in the shape of the trade-off between larval density and average larval mass more quantitatively, using our preliminary analyses to choose appropriate regression models to compare individuals in the Full Care and No Care treatments. We found significant interactions between the presence of care and larval density, and the presence of care and larval density^2^ in models excluding and including the experimentally reduced broods (Table 2). These significant interactions more formally demonstrate that the shape of the relationship between larval density and average larval mass differed between the Full Care and No Care treatments.

**Table 2:** Results from GLMs examining the effects of parental care, larval density, larval density^2^, larval density^3^, and interactions between the parental care and larval density terms on average larval mass in the first generation of the experiment

	Excluding manipulated broods		Including manipulated broods	
Factor	*F*_1, 249_	*P*	*F*_1, 287_	*P*
Care	2.74	0.099	112.06	<0.0001
Density	3.28	0.071	4.81	0.029
Density^2^	12.33	0.00053	2.29	0.13
Density^3^	14.31	0.00019	7.46	0.0067
Care × density	4.42	0.037	57.65	<0.0001
Care × density^2^	4.47	0.035	32.81	<0.0001

We present the results for models excluding and including experimentally reduced broods.

### EXPERIMENT 2: THE EFFECT OF LARVAL DENSITY ON THE ABILITY OF LARVAE TO ENTER THE CARCASS AND CARCASS DECOMPOSITION

Larval density had a large effect on the ability of larvae to penetrate the breeding carcass in the absence of parents. In the low-density treatment, larvae were unable to penetrate the breeding carcass (0/21 replicates had larvae feeding within the carcass 90 h after pairing). However in the high-density treatment, 35% of the replicates (8/23) had larvae feeding within the carcass 90 h after pairing. The difference between these two proportions is significant (χ^2^ = 6.74, *P* = 0.0094).

There was no evidence that larval density influenced the growth of mold on the carcass. At 90 h postpairing, 38% (8/21) of the low-density replicates had mold growing on the carcass compared with 61% (14/23) of the high-density replicates. The difference between these two proportions is not significant (χ^2^ = 1.46, *P* = 0.23). At 114 h postpairing, 52% (11/21) of the low-density replicates had mold growing on the carcass and 61% (14/23) of the high-density replicates had mold growing on the carcass. The difference between these two proportions is not significant (χ^2^ = 0.069, *P* = 0.79).

**Figure 2:**
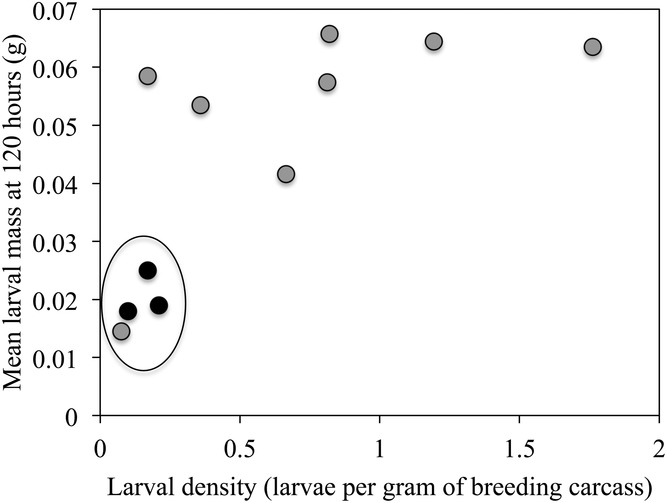
The relationship between larval density and mean larval mass in experimental low-density (black) and high-density (gray) broods. Circled broods were feeding outside the carcass. All other broods were feeding within the carcass.

The above results suggest that the ability of larvae to penetrate the breeding carcass, and feed upon the flesh within, increased with larval density. But the key question to answer next is: does penetrating the carcass improve larval fitness? To answer this question, we examined whether larvae feeding within the carcass had a greater mass than those feeding outside the carcass, and found this was indeed the case (Wilcoxon test comparing larval mass between broods feeding inside and outside the carcass, *W* = 28, *P* < 0.0061). Furthermore, there was a positive correlation between larval density and average larval mass at this stage (Spearman's correlation, *r* = 0.81, *P* = 0.0044). We note that the range of densities across which larval mass increased in experiments 1 and 2 is very similar (compare Figs.1 and 2).

## Discussion

Here we have shown that the balance between cooperation and competition among burying beetle larvae is influenced by the presence of posthatching parental care and larval density. When parents were allowed to provision young there was a strong negative relationship between larval density and average larval mass. In the absence of parental care, initial increases in larval density had beneficial effects on average larval mass but further increases in larval density reduced larval mass. This nonlinear relationship between larval density and larval mass likely reflects a density-dependent shift between mutually beneficial and competitive larval interactions.

Our experiment is similar to a previous study that manipulated both the presence of posthatching parental care and brood size in *N. vespilloides* (Smiseth et al. 2007). In this previous study Smiseth et al. (2007) found that when parents were allowed to care for their young, there was a negative impact of brood size on larval growth but this impact disappeared in the absence of parental care, just as we report here. However in Smiseth et al.'s (2007) study, the relationship between brood size and larval growth was flat in the absence of care whereas we found a hump-shaped relationship between larval density and average larval mass in the absence of parental care. Three key methodological differences between our experiment and Smiseth et al.'s likely explain this difference. First, Smiseth et al. (2007) manipulated broods to contain 5, 20, or 40 larvae. In contrast, we used continuous variation in brood size with broods containing 1 to 44 larvae (mean brood size = 15.54 larvae). Therefore the increase in larval mass with larval density that we observed occurred across a range of brood sizes that were generally smaller than those included in Smiseth et al.'s (2007) experiment. Thus, Smiseth et al.'s experiment could not have detected the dynamics that we observed. Second, Smiseth et al.'s experiment involved removing parents from the breeding carcass at larval hatching whereas ours involved removing parents nearly 24 h before hatching. During the 24 h, between the completion of egg laying and hatching, parents prepare a shallow cavity in the carcass in which larvae seemingly congregate to feed from after hatching. In our experiment this component of prehatching parental care was eliminated whereas in Smiseth et al.'s it was not. As a result, the larvae in our experiment had to penetrate the carcass without their parents help. Third, Smiseth et al. (2007) used breeding carcasses that were nearly twice as heavy as the ones used in our experiment. All of the resources that fuel larval development in *N. vespilloides* are contained within the breeding carcass, thus the size of the breeding carcass will influence the level of sibling competition (Smiseth et al. 2014). It is possible that the carcass sizes used by Smiseth et al. (2007) minimized sibling competition in the absence of parental care, whereas the carcass sizes used in our study induced sibling competition in relatively large broods.

The decline in larval mass with larval density we observed in the Full Care treatment is consistent with the scenario described by Smiseth et al. (2007) wherein increasing larval density reduces the ability of individual larvae to effectively solicit food from their parents. How, though, might we explain the nonlinear relationship between larval mass and larval density we observed in the No Care treatment? We suggest two hypotheses to explain this pattern. First, the ability of a brood to penetrate and use the breeding carcass might increase with larval density, perhaps because there are simply more mouths on the carcass. However, the benefits of having many mouths on the carcass may reach an asymptote beyond which increasing larval density results in exploitative competition between siblings for a fixed pool of resources. A second possibility is that the benefits of social immunity (Cotter and Kilner 2010a), mediated through the production of antimicrobial exudates, are density dependent. *Nicrophorus vespilloides* parents smear the breeding carcass with antimicrobial exudates (Cotter and Kilner 2010b) that delay decomposition of the carcass (Arce et al. 2012, 2013). Larvae also produce these exudates (Arce et al. 2013; Reavey et al. 2014) and thus, larval density increases, the social benefits of exudate production might also increase. However, beyond a certain larval density the nutritional impact of exploitative sibling competition might overwhelm the effects of social immunity.

The results of our second experiment are more consistent with the “many mouths” hypothesis than with the social immunity hypothesis. Specifically, we found that high-density broods were much more likely to chew their way into the breeding carcass than low-density broods and that, although our sample size was quite small, feeding within the carcass always resulted in heavier larvae compared with feeding outside the carcass (Fig.2). However, there were no significant differences between the high- and low-density treatments in the proportion of carcasses with evidence of decomposition (i.e., mold). Nevertheless, our measure of decomposition was crude and focused only on fungi. It is possible that larval density affects bacterial growth. It is also possible that any communal benefits that larvae gain through their production of antimicrobials are evident sooner than we sampled because the potency of the larval antimicrobials peaks 24 h after hatching (Reavey et al. 2014).

Although posthatching parental care is facultative in *N. vespilloides*, breeding success and larval performance are improved by its provision (Eggert et al. 1998). This suggests that the absence of posthatching care creates a stressful environment for developing larvae. Intriguingly our results suggest that stressful conditions (i.e., the absence of posthatching care) facilitate mutually beneficial interactions between larvae whereas benign conditions (i.e., the presence of posthatching care) facilitate competition (see also Smiseth et al. 2007). This result runs counter to the observation that sibling competition is often more intense in stressful environments (e.g., low resource environments) than in more benign environments (e.g., high resource environments; Mock and Parker 1997) but is concordant with the results of a recent study showing that European earwig nymphs spend more time engaged in cooperative behaviors when their mothers are absent than when they are present (Falk et al. 2014). There is a well-known taxonomic bias in the study of sibling interactions, with much of the focus on avian families in which care is obligate and offspring are not capable of self-feeding, conditions that are likely to intensify sibling competition (Mock and Parker 1997; Roulin and Dreiss 2012). Our results suggest that facultative parental care allows a more diverse range of sibling interactions to exist and that the balance between competition and cooperation is sensitive to environmental pressures.

## DATA ARCHIVING

The doi for our data is 10.5061/dryad.qt4nf.
